# Spray-Dried Inhalable Powder Formulations of Gentamicin Designed for Pneumonic Plague Therapy in a Mouse Model

**DOI:** 10.3390/pharmaceutics14122646

**Published:** 2022-11-29

**Authors:** Menghuan Zhu, Dongna Zhang, Lili Zhang, Liangliang Zhao, Likun Xu, Baogang Wang, Xinyu Zhang, Jinwei Chen, Zhuchun Bei, Hong Wang, Dongsheng Zhou, Wenhui Yang, Yabin Song

**Affiliations:** 1 School of Public Health and Health Management, Gannan Medical University, Ganzhou 341000, China; 2 State Key Laboratory of Pathogen and Biosecurity, Beijing Institute of Microbiology and Epidemiology, Beijing 100071, China

**Keywords:** pulmonary drug delivery, gentamicin, spray-drying technology, formulation, *Y. pestis*, therapeutic effect

## Abstract

Infection with Yersinia pestis (*Y. pestis*) may cause pneumonic plague, which is inevitably fatal without treatment. Gentamicin (GM), an aminoglycoside antibiotic, is a drug commonly used in the treatment of plague. However, it requires repeated intramuscular or intravenous administration. Pulmonary drug delivery is noninvasive, with the advantages of local targeting and reduced risk of systemic toxicity. In this study, GM powders were prepared using spray-drying technology. The powders displayed good physical and chemical properties and met the requirements for human pulmonary inhalation. The formulation of the powders was optimized using a 3^2^ full factorial design. A formulation of 15% (*w/w*) of L-leucine was prepared, and the spray-drying process parameters using an inlet temperature of 120°C and a 15% pump rate were determined to produce the best powder. In addition, the optimized GM spray-dried powders were characterized in terms of morphology, crystallinity, powder fluidity, and aerodynamic particle size distribution analysis. In a mouse model of pneumonic plague, we compared the therapeutic effects among three administration routes, including subcutaneous injection, liquid atomization, and dry powder atomization. In conclusion, our data suggest that inhalation therapy with GM spray-dried powders is an effective treatment for pneumonic plague.

## 1. Introduction

The Gram-negative bacterium *Y. pestis* is a zoonotic bacterial pathogen that has caused devastating pandemics, resulting in the deaths of an estimated 200 million people throughout history [1]. With improvements in living conditions and public health, the incidence of plague outbreaks has gradually decreased [2]. However, outbreaks and sporadic cases continue to occur [3]. In recent years, cases of plague have been reported in the Democratic Republic of the Congo, Madagascar, and Peru [4,5]. The worst plague epidemic of the 21st century in Madagascar resulted in approximately 2400 cases and 200 deaths, causing global concern [6]. Along with remaining a threat in endemic areas, *Y. pestis* is also classified as a Tier 1 category A agent (highest bioterrorism risk) because of its potential use as a respiratory-transmitted biological weapon, which makes it a serious threat to public health and safety [7].

Bubonic plague, septicemic plague, and pneumonic plague are the three main clinical forms of this disease [8]. The inhalation of *Y. pestis* results in the most severe form of the disease, pneumonic plague. If left untreated, death can occur in two to three days or even within hours, and mortality can reach 100% [9,10]. The main clinical symptoms of patients with pneumonic plague include fever, breathing difficulties, chest pain, pulmonary insufficiency, hemoptysis, and sepsis [11,12]. Although the plague develops rapidly and carries a high fatality rate, it can be successfully treated with antimicrobials [13]. Antibiotics currently approved by the Food and Drug Administration (FDA) for the treatment of plague include streptomycin, tetracyclines, and fluoroquinolones [14]. From 1985 to 1999, 18 cases of plague in New Mexico were treated with GM or GM plus tetracycline with positive results [15]. In 2002, a randomized controlled trial of GM and doxycycline for plague was conducted in Tanzania, in which 35 patients were treated with GM, and only 2 deaths (6%) occurred on the first day of treatment [16]. Since then, GM is recognized as an effective alternative to streptomycin and has become the first-line treatment for plague in the United States [17].

The pulmonary delivery of antibiotics to treat pulmonary disease provides many advantages compared to conventional parenteral and oral routes. Pulmonary delivery helps to shorten the treatment time and reduce lung damage caused by *Y. pestis*, enabling rapid reduction of the lung bacterial load and reducing the risk of secondary infection [18,19,20]. Gur et al. [10] found that the inhalation of GM accelerated the clearance of *Y. pestis* bacteria from the lungs of infected animals and could be used as a treatment for pneumonic plague. The main pulmonary inhalation formulations include metered dose inhalers, dry powder inhalation, sprays, and inhalation solutions [21]. Among these, dry powder inhalation is an attractive alternative to deliver drugs directly to the lungs, as it is not limited by drug solubility issues and is regarded as being more stable than liquid dosage forms [22].

The formulation and delivery process have encountered several challenges due to the hygroscopic character of GM components [23]. In order to make the powder suitable for inhalation, excipients are used to reduce the hygroscopicity and to enhance the powder flow properties. For example, L-leucine (Leu) was added to the formulation as a dispersibility enhancer to improve the aerosol properties of the powder [24,25,26]. It was shown to improve the aerosolization performance of powders by reducing hygroscopicity, increasing dispersibility, or altering surface morphologies [24]. Therefore, Leu was also chosen as the excipient in the spray-dried powders for inhalation in our experiment.

In this study, we report the preparation and evaluation of a dry powder of GM for topical delivery to the lungs, which could potentially be used as a treatment for pneumonic plague. To optimize the aerodynamic properties of the spray-dried powders, we investigated the excipient concentration as well as the spray-drying conditions. Pharmacokinetic studies were performed in a mouse model to assess the disposition of GM after inhalation of the dry powder in the lungs.

## 2. Materials and Methods

### 2.1. Materials

GM and Leu were supplied by Energy Chemical. O-phthalaldehyde, KOH, sodium L-heptanesulfonate, boric acid, mercapto acetic acid, glacial acetic acid (for analysis, USP grade), isopropanol alcohol (for analysis, USP grade), and methanol (for analysis, USP grade) were supplied by Shanghai Titan Scientific Co., Ltd., Shanghai, China. Size #3 gelatin capsules were gifted by Suzhou Singmed Medical Device Science and Technology Ltd., Suzhou, China.

### 2.2. Preparation of Powder Formulations

Micronized particles of GM alone or with Leu were prepared by spray-drying 4:1 (*v/v*) distilled water–isopropyl alcohol solvent mixtures. Briefly, GM and Leu were both dissolved in water, and then, isopropanol alcohol was added under continuous stirring, reaching a total concentration of 2.5% (*w/v*). Each solution was spray-dried using the Mini Spray Dryer B-290 (BUCHI Labortechnik AG, Flawil, Switzerland) at three different inlet temperatures. The drying air flow was 667 L/h (40 mm), and the aspiration rate was 35 m^3^/h (100%). Each preparation was carried out in triplicate. The GM spray-dried powders were prepared using a 3^2^ randomized full factorial design. The Leu content (A), inlet temperature (B), and pump rate (C) were selected as the independent variables, while parameters Y = d50 ∗ 0.5/minimum − yield ∗ 0.5/maximum yield, where d50 is the particle size at which 50% of the particles are smaller, were selected as the dependent variables (response parameters). The independent factors (coded and their actual values) and their combinations are shown in Table 1.

### 2.3. Scanning Electron Microscopy (SEM)

The morphology of the spray-dried powders was analyzed by SEM (Hitachi S-3400N, Hitachi Limited, Tokyo, Japan) [27]. The SEM analysis was performed with an S-3400 field emission scanning electron microscope with an accelerating voltage of 20 kV. The powder particles were coated with a gold layer that imparted sufficient conductivity to the particles before being investigated.

### 2.4. Particle Size

The diameter and distribution of the spray-dried powders were measured by a laser particle sizer (RODOS & HELOS, Sympatec, Clausthal-Zellerfeld, Germany) using a dry method [28]. Particle size distribution is typically represented as d10, d50, and d90, which represent the percentage of particles below a given size (µm) [29]. In addition, the term “span” is defined as (d90 − d10)/d50 to describe the width of the size distribution [30]. All samples were tested in triplicate and were expressed as means ± standard deviations (SD).

### 2.5. Differential Scanning Calorimetry (DSC)

The thermal behavior of the GM raw material, Leu raw material, a physical mixture of the GM and Leu raw materials, and GM spray-dried powders was identified by DSC using a DSC 6000 (PerkinElmer, Waltham, MA, USA). The samples were placed in a perforated aluminum pan, the temperature was raised at 10 °C/min to 500 °C, and the DSC curve of each sample was recorded [31].

### 2.6. X-ray Powder Diffraction (XRPD)

The crystalline behavior of the samples was tested by using a Bruker D8 diffractometer (Bruker AXS GmbH, Brooke, Germany). Samples were placed on a horizontal quartz glass holder plate and irradiated with Cu Kα radiation (45 kV, 40 mA) with a 2θ scan range of 10–80° at a rate of 10°/min at 25 °C in the uniform scan mode [32].

### 2.7. Density and Fluidity

The Carr’s Index (CI), Hausner ratio (HR), and angle of repose (θ) parameters were used to estimate the flow properties of the powder samples for drug delivery [33]. These were calculated from the bulk density (ρbulk) and tapped density (ρtap) using the equation CI = (1 − ρbulk/ρtap)∗100% and Hausner ratio = 100/(100 − CI) [34]. Bulk and tapped densities were measured by using a Copley Tapped Density Tester (Copley Scientific Limited, Nottingham, United Kingdom) [35]. The angle of repose was measured by using the fixed funnel technique, in which the powder was poured onto a flat horizontal surface through a funnel to form a conical pile with an angle θ [36].

### 2.8. Hygroscopicity and Hygroscopic Curve

Moisture sorption plays a key role in dry powder characteristics and stability, which can affect the powder aerosolization efficiency and subsequent lung deposition [37,38,39]. Seven samples of the same mass of drug were weighed into a glass desiccator containing a concentrated sulfuric acid solution and different saturated salt solutions and then maintained in a constant temperature chamber at 25 °C for 96 h [40]. The weighing bottles were removed and weighed precisely, and the moisture absorption rate was calculated, and a moisture absorption curve was drawn.

### 2.9. High-Performance Liquid Chromatography (HPLC)

The content of GM was determined through analysis with an Agilent 1100 series HPLC system (Agilent Technologies Co., Ltd., Wilmington, NC, USA) at a wavelength of 330 nm on an XBridge^®^ C18 (4.6 × 250 mm, 5 μm) column. All samples were pretreated prior to testing using the following method: 440 μL of isopropanol and 160 μL of O-phthaldialdehyde reagent (OPA) were added to each 400 μL sample for derivatization [23,41,42]. Then, each sample was mixed for 10 ± 1 s, heated in an oven at 60 ± 3 °C for 15 ± 1 min, cooled at room temperature, filtered through 0.45-µm filters, and analyzed by HPLC [43]. We conducted a linearity study of the standard curve using GM concentrations between 50 µg/mL and 500 µg/mL with an injection volume of 10 μL.

### 2.10. Aerodynamic Particle Size Distribution Analysis

In vitro deposition and aerodynamic particle size were determined by using a Next-Generation Pharmaceutical Impactor (NGI) (Beijing HuiRongHe Technology Co., Ltd., Beijing, China) [34,44]. Approximately 30 mg of the powder was inserted into a #3 HPMC capsule and dispersed by a HandiHaler^®^ dry powder inhaler (DPI) into the NGI with a total 8-L volume of airflow. The flow rate of the instrument was set as 30 ± 5 L/min, and the inhalation time was set as 10 s. Particle bounce was avoided by coating the collection cups with silicon oil before each measurement [45]. After dispersal, the powders in the impactor were leached using water, and then, the washing solution was collected and analyzed by HPLC. Each sample was tested in triplicate. The mass median aerodynamic diameter (MMAD), geometric standard deviation (GSD), and fine particle fraction (FPF) were calculated using Excel and CITDAS software [46,47].

### 2.11. Pharmacodynamic Study In Vivo

#### 2.11.1. Animals

Female BALB/c mice (SPF) 6–8 weeks old were obtained from Beijing Vital River Laboratory Animal Technology Co., Ltd. (Beijing, China) and were used for all experiments. The mice had free access to food and water throughout the course of the study. This study was approved by the Institute of Animal Care and Use Committee (IACUC) at the Academy of Military Medical Sciences (AMMS). Mice were allowed to acclimatize to their home cage environment for 1 week before the challenge.

#### 2.11.2. Bacteria Strain

*Y. pestis* strain 201is maintained in our laboratory. This strain belongs to a newly established *Y. pestis* biovar *Microtus*, which is widely believed to be highly lethal to mice but avirulent to larger mammals, including guinea pigs, rabbits, nonhuman primates, and humans [48]. Strain 201 has a LD50 of 3 CFU, 1.9 CFU, and 20 CFU for BALB/c mice by the subcutaneous, intravenous, and pulmonary delivery routes, respectively [28,49]. Strain 201 was cultivated in brain heart infusion broth (BHI; BD, Voigt Global Distribution Inc., Lawrence, KS; dilution 1:20) at 26 °C in a shaking incubator at 220 rpm to an optical density at 600 nm (OD600) of approximately 1.0. Cultures were then inoculated with BHI (dilution 1:100) and maintained at 26 °C and again cultured to an OD600 of 1.0. After that, cultures were transferred to a 37 °C shaking incubator for another 3 h. Colony counts were determined by serial dilution and plated on 5% sheep blood agar plates (Luqiao, Beijing, China). The plates were incubated for 3 d at 26 °C, and the colonies were counted.

#### 2.11.3. Animal Administration and Treatment

One hundred and fifty BALB/c mice were randomly divided into five groups: a normal control group, infection model group, subcutaneous injection group, liquid atomization group, and dry powder atomization group. Except for the normal control group, mice were anesthetized by intraperitoneal injection of pentobarbital sodium (70 mg/kg of body weight) and challenged with 1000 CFU/mice of *Y. pestis* strain 201 by a pulmonary delivery route. After a 1-h challenge, the subcutaneous injection group was injected following a regimen of 20 mg/kg·d, the liquid atomization group was atomized with a 3.5 mg/kg·d GM injection, and the dry powder atomization group was atomized with 4.2 mg/kg·d GM spray-dried powders (active ingredient 3.5 mg/kg·d). The experimental groups were treated with drugs for 5 d. The normal control group was treated with sterile normal saline in the same manner and moved to another room for feeding.

#### 2.11.4. Clinical Signs and Survival Rate

Ten mice were observed for 14 d post-challenge, and clinical signs (furring, dyspnea, forced abdominal breathing, and retarded response to touch or external stimulation) were recorded twice daily. The number of dead mice was recorded, and the survival curves were plotted.

#### 2.11.5. Bacterial Count

At different time points (0 h, 24 h, 48 h, and 72 h) after administration, three mice in each group were sacrificed to test the bacterial load. About 100 mg of the liver, spleen, and lungs were homogenized in 800 µL PBS. Then, 50 µL of the homogenate sample was diluted in PBS, and 10 µL of diluent was inoculated on blood agar plates for bacterial counting [50].

#### 2.11.6. Histopathology

Part of the lungs, liver, and spleen of the mice were collected at 0 and 5 d after treatment administration. Each organ was immediately placed in 4% paraformaldehyde for 24 h prior to processing. Prior to evaluation, tissue sections were stained with hematoxylin and eosin (HE). The pathological changes in the tissue sections were observed by light microscopy. Tissue sections were evaluated by a trained pathologist blind to the treatment according to the following scores: 0, no pathological lesions; 1, minimal; 2, mild; 3, moderate; and 4, severe.

### 2.12. Statistical Analysis

Data were expressed as the mean ± standard deviation. All statistical analyses were performed using SAS statistical software (version 9.1, SAS Institute Inc., Cary, NC, USA) or GraphPad Prism 8.0.

## 3. Results

### 3.1. Optimization of Spray-Drying Process Parameters

The product yield of spray-drying was affected by the Leu content in the formulation, inlet temperature, and pump rate (Table 2). The results of the ANOVA are shown in Table 3. The order of influence of the three factors on the evaluation index was: Leu content > pump rate > inlet temperature, in which the Leu content had a significant influence. The best parameter combination was A3B2C2; 15% (*w/w*) of Leu spray-dried at an inlet temperature of 120 °C and a 15% pump rate. Three batches of GM spray-dried powders were prepared according to the optimized parameters. The average yields of the three batches of powder were 85.40 ± 2.30%, 86.12 ± 1.02%, and 86.27 ± 0.88% (*n* = 3); the d50 values were 3.87 ± 0.63 μm, 3.70 ± 0.72 μm, and 3.90 ± 0.85 μm (*n* = 3); and the span was 1.93 ± 0.12 μm, 2.10 ± 0.10 μm, and 1.89 ± 0.21 μm (*n* = 3). This indicated that the process was reproducible and could be used for the preparation of GM spray-dried powders.

### 3.2. Physicochemical Properties of the Optimized GM Spray-Dried Powders

#### 3.2.1. Particle Morphology

**Figure 1:**
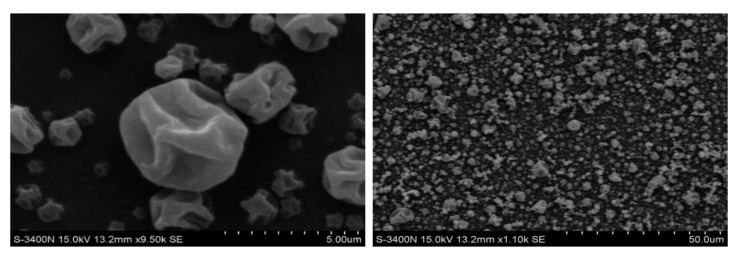
The scanning electron microscope (SEM) images of spray-dried particles.

As shown in Figure 1, the particles prepared by the co-spray-drying of GM and Leu became raisin-like and were irregularly wrinkled. This was because of the relative hydrophobicity and surfactant properties of Leu. In addition, the low solubility of Leu allowed it to rapidly reach a supersaturated state at the beginning of spray-drying, resulting in the formation of a hydrophobic shell on the surface of the dried droplets. During drying, Leu on the droplet surface shrank rapidly as the solvent evaporated, leading to the particle collapse to form a wrinkled outer surface.

#### 3.2.2. Thermal Analysis and Powder Crystallinity

**Figure 2:**
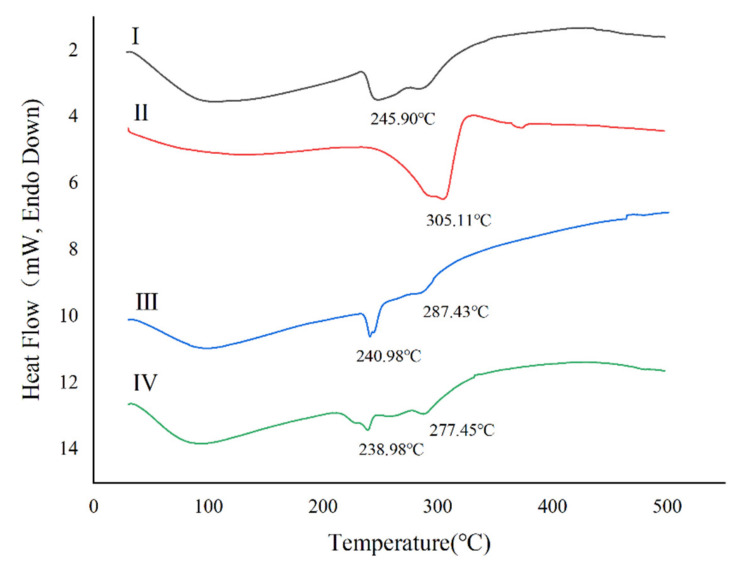
Differential scanning calorimetry (DSC) thermograms of (**I**) GM raw material. (**II**) Leu raw material. (**III**) Physical mixture of GM and Leu powder raw materials. (**IV**) GM spray-dried powders.

**Figure 3:**
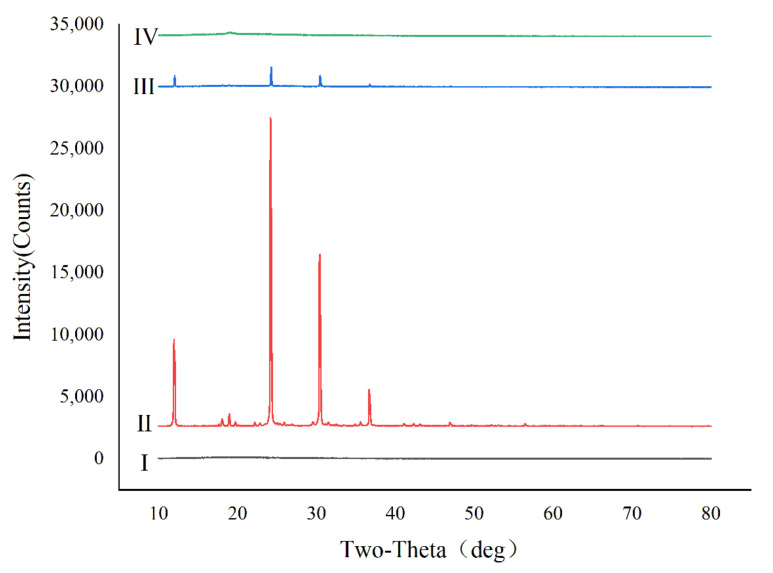
X-ray powder diffraction (XRPD) of (**I**) GM raw material, (**II**) Leu raw material, (**III**) physical mixture of GM and Leu powder raw materials, and (**IV**) GM spray-dried powders.

The recorded thermograms of the GM raw material, Leu raw material, physical mixture of GM and Leu raw materials, and GM spray-dried powders were investigated using DSC (Figure 2). The results showed a melting endothermic peak at 245.90 °C in the curve of GM (Figure 2I) and a melting endothermic peak at 305.11 °C in the curve of Leu (Figure 2II). The curves of the GM raw material and Leu mechanically mixed powder (Figure 2III) showed two melting endothermic peaks, which were 240.98 °C and 287.43 °C. The DSC curve of the mechanical mixture was basically a simple superposition of the corresponding curves of the GM and Leu raw materials. The GM spray-dried powders (Figure 2IV) exhibited a similar DSC thermogram as the physical mixture, showing two endothermic peaks at 238.98 °C and 277.45 °C belonging to GM and Leu, respectively. The further reduction of the endothermic peak temperature of GM in the spray-dried powders was attributed to an interaction of GM and Leu. The crystallinity of the GM raw material, Leu raw material, physical mixture of GM and Leu raw materials, and GM spray-dried powders were also investigated by XRPD. As shown in Figure 3, the GM raw material was in an amorphous state, while the Leu excipient was in a crystalline state. The physical mixture of the two showed the characteristic crystal peak of Leu, but the spray-dried drug showed no crystal diffraction peak, indicating that GM mainly existed in an amorphous form.

#### 3.2.3. The Tapped Density, Fluidity, and Hygroscopicity

The bulk and tapped density of GM spray-dried powders were 0.364 ± 0.032 g/cm^3^ and 0.512 ± 0.031 g/cm^3^, respectively. Accordingly, the CI and Hausner ratio were calculated as 28.558 ± 0.086 and 1.003 ± 0.001, respectively. In addition, the angle of repose (θ) value was 38.4°. The CI values < 25 indicated good flowability and values higher than 40 related to poor flow characteristics. The low Hausner ratio also indicated a more desirable flow ability. The small repose angle indicated that the powder had low friction and good flowability. Based on this, the powder showed acceptable flow properties. Humidity control is also important in the production and storage process. In our experiment, the critical relative humidity of the GM spray-dried powders was 56%, which is favorable for the production and storage of the powder.

#### 3.2.4. In Vitro Lung Deposition Rate

**Figure 4:**
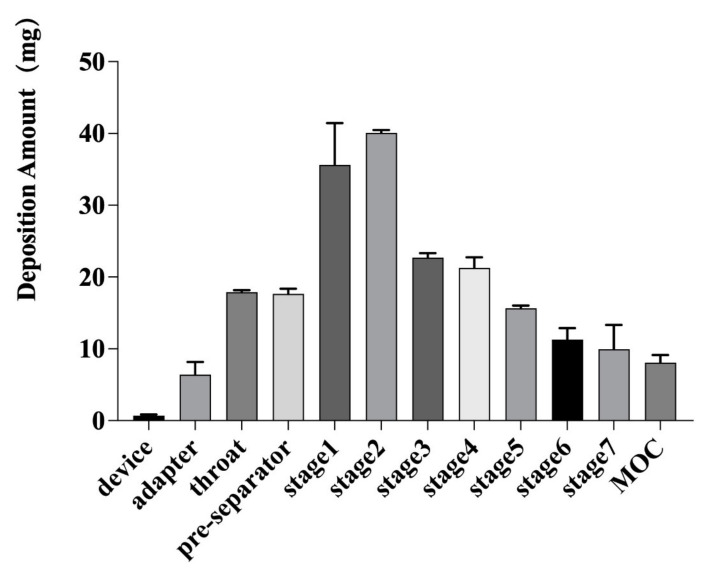
Dissemination amount of the GM spray-dried powders from each part of the NGI (device (HandiHaler^®^ DPI, Boehringer Ingelheim Pharm GmbH&Co. KG, Ingelheim, Germany), adapter, throat, pre-separator, stages 1–7, and micro-orifice collector (MOC)) (*n* = 3). Cutoff sizes for the NGI at a flow rate of 30 L/min were 11.7, 6.4, 3.99, 2.3, 1.36, 0.83, and 0.54 μm for stages 1–7 and the MOC, respectively [51].

The effective deposition rate and MMAD of the prepared GM spray-dried powders were determined according to the method described above. The aerodynamic characteristics of the GM spray-dried powders were evaluated using an NGI and are presented in Figure 4. The results showed that the MMAD, GSD, and FPF% of the GM spray-dried powders were 4.94 ± 0.45 µm, 3.62 ±0.03, and 42.36 ± 0.42%, respectively, indicating that the powder was suitable for pulmonary inhalation.

### 3.3. GM Spray-Dried Powders Effectively Protected Mice Infected with Y. pestis

#### 3.3.1. Protection Efficacy after Aerosol Challenge with 50× LD50 of *Y. pestis* Strain 201

After observing and recording the control and treated mice symptoms, the normal control group showed no obvious symptoms. The hair of the mice in the infection model group became erect on the second day after the challenge. On the third day, some mice showed symptoms, such as secretion at the corners of the eyes and a sluggish response after external stimulation. The symptoms of the infection model group mice gradually worsened, and forced abdominal breathing was visible. The subcutaneous injection group and the liquid atomization group had no symptoms. However, the hair of the mice in the dry powder atomization group was slightly erect because of a stress response that was induced by lung irritation caused by the presence of large particles of the powders. This symptom gradually disappeared after the completion of dry powder atomization.

**Figure 5:**
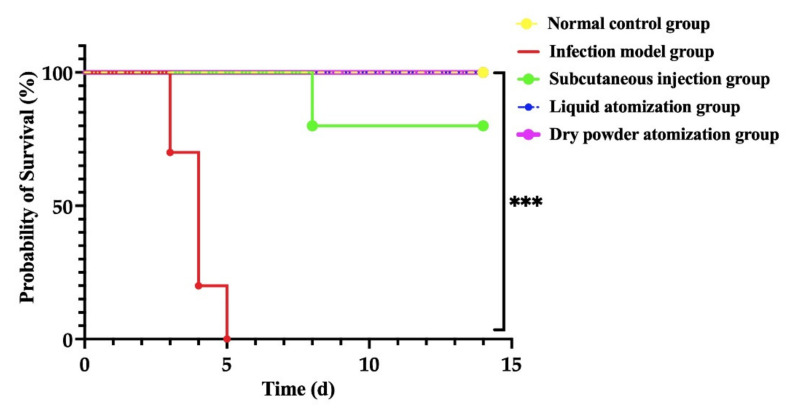
Survival curves for mice challenged with aerosolized *Y. pestis* strain 201. Mortality data were analyzed using the Kaplan–Meier survival analysis. *** *p* < 0.001.

As shown in Figure 5, a total of 10 mice died in the infection model group with a mortality rate of 100%, which was significantly higher than the mortality rate of the normal control group (*p* < 0.001). The average survival days of mice in the infection model group was 3.9 d, and the average survival time was significantly shorter (*p* < 0.001) compared with the normal control group. In contrast, the animal mortality of the subcutaneous injection group, liquid atomization group, and dry powder atomization group decreased significantly (*p* < 0.001). Compared with the subcutaneous injection group, the survival time of the liquid atomization group and dry powder atomization group was prolonged, but there was no significant difference (*p* > 0.05). The dry powder atomization group mice were given GM spray-dried powders 1 h after the aerosol challenge with the *Y. pestis* strain, which significantly prolonged the survival time and reduced the mortality rate. Therefore, the GM powder showed obvious protective effects in vivo. The therapeutic effects of the 3.5 mg/kg·d inhaled dry powder dosage were better than the effects of the 20 mg/kg·d of injected GM.

#### 3.3.2. Bacterial Enumeration from Mouse Organs after Administration of GM

**Figure 6:**
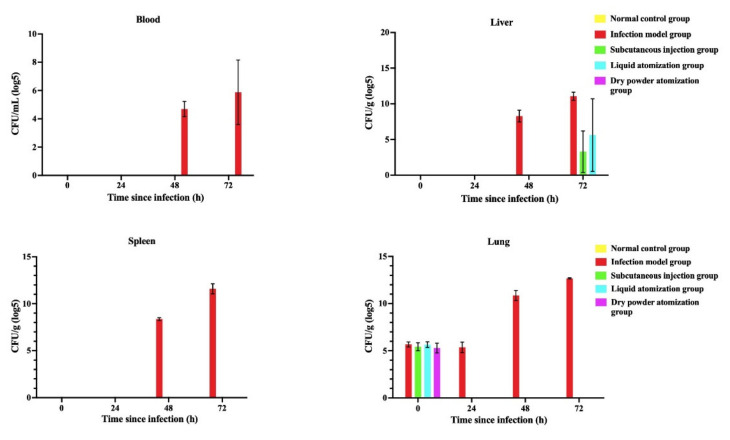
Bacterial loads in the blood, liver, spleen, and lungs of mice at different sampling time points.

Bacterial loads in the blood, liver, spleen, and lungs of mice at different sampling time points were investigated, and the results are shown in Figure 6. In the normal control group, no bacteria were detected in the blood or organs of mice. The amount of bacteria in the blood and organs of mice in the infected model group showed an increasing trend over time, indicating that the bacteria continued to multiply in the mice after infection. Plague bacteria were detected in the livers of mice in the subcutaneous injection group and liquid atomization group at 72 h but not in the dry power atomization group. This suggests that GM spray-dried powders effectively inhibited the pestis bacteria in the blood, liver, spleen, and lungs of mice after mice were challenged by *Y. pestis* strain 201 for 1 h.

#### 3.3.3. Histopathological Analysis of Mouse Tissues after Immunization and Challenge

**Figure 7:**
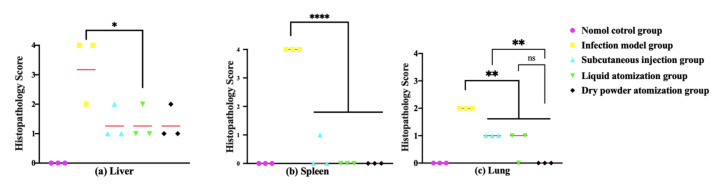
Pathological lesions in the tissues of mice euthanized 5 d after the *Y. pestis* strain 201 intratracheal challenge. Tissue from lungs, livers, and spleens were collected from three mice per group, fixed in formalin, embedded in paraffin, and stained with HE. Pathological scores of (**a**) liver tissue, (**b**) spleen tissue, and (**c**) lung tissue are presented. Tissue sections were evaluated by a trained pathologist according to the following scores: 0, no pathological lesions; 1, minimal; 2, mild; 3, moderate; and 4, severe. The degree of pathological lesions was related to the distribution and severity of the lesions as follows: (I) edema and (II) tissue parenchymatous lesions, such as congestion and hemorrhage. Data are expressed as the mean ± SD (*n* = 3) for data collected from one representative experiment. * *p* < 0.05; ** *p* < 0.01; **** *p* < 0.001.

In the infection model group, perivascular edema, neutrophil infiltration, and hemorrhage were observed in the lungs, and inflammatory cells were recruited into the white pulp of the liver and spleen. The pathological scores of the liver, spleen, and lungs in each drug treatment group were significantly lower than those in the infection model group (Figure 7), indicating that the administration of GM had appreciable protective effects against *Y. pestis*. The lung score of the dry powder atomization group was significantly lower than that of the subcutaneous injection group (*p* < 0.01). Furthermore, the GM spray-dried powder treatment resulted in rapid clearance of bacteria from the lungs, potentially improving the condition of the lungs after recovery and reducing the risk of secondary infections. These results suggest that GM spray-dried powders (3.5 mg/kg·d) has better efficacy than subcutaneous injections (20 mg/kg·d). Compared with subcutaneous injections, the dose of spray-dried powders was lower and still effectively inhibited the pestis bacteria in the blood, liver, spleen, and lungs.

## 4. Discussion

Although the plague is no longer a global pandemic, it is still prevalent in some areas. Additionally, *Y. pestis* is classified as a Class 1 bioterrorism select agent, and it poses a serious threat to public health and safety [52]. Therefore, there is still a need to develop improved therapeutic approaches against *Y. pestis*. The plague primarily invades humans through the respiratory tract and targets the human lungs with a rapid onset. Emergency prophylaxis and treatment of exposed people before or during the early stages of the disease is important to improve the survival rate and reduce casualties. Traditional treatment methods, such as oral drugs, need to pass through the digestive tract to reach the bloodstream to take effect, so the effect is always slow. Although intravenous injections can act quickly, they cannot meet the needs of large numbers of people in a short period of time during wartime due to the need for specialized medical personnel. The basic principle of inhaled antibiotics for the treatment of pulmonary infections is extremely simple: the drug is delivered directly into the lungs to achieve higher local concentrations at lower systemic doses while reducing systemic exposure, thereby reducing adverse effects [53].

In this study, we investigated the spray-drying process conditions and prepared a powder containing GM and the excipient Leu. Both the Leu content in the formulation and spray-drying conditions influenced the production yield and the particle size. When the inlet temperature was low, drying was incomplete, leading to the adherence of droplets on the inner surfaces of the cyclone and, hence, a low production yield [54]. The production yield was the highest at an inlet temperature of 120 °C, above which the yield started to decline. The inlet temperature also influenced the particle size and size distribution of the spray-dried powders. At an inlet temperature of 120 °C, the microparticulate aerosols were successfully produced with a narrow particle size distribution and suitable particle shape. When pump rate was low, the spray-drying time was long, and the particle size was large. At a higher pump rate, the particle size was small. However, due to insufficient contact with hot air, the sticky wall phenomenon readily occurred, resulting in a low yield.

The addition of Leu has been shown to cause particles to gain a raisin-like appearance that is irregularly shaped with a wrinkled surface [55]. Using Leu in the current study as an excipient resulted in wrinkled particles with irregularly dimpled shapes (Figure 1), consistent with the known spray-drying effects of Leu. The shape has been shown to be beneficial for particles intended for inhalation. Because the corrugated surfaces improve the powder dispersibility by minimizing the contact area and reducing interparticle cohesion, and therefore, corrugated particles are better dispersed than spherical particles [56].

We also investigated the therapeutic effect on plague in a mouse model. The therapeutic effects in the infection model group, subcutaneous injection group, liquid atomization group, and dry powder atomization group were compared. The nebulized administration of GM spray-dried powders, such as the subcutaneous injection and liquid nebulized inhalation, reduced the bacterial burden in the blood, liver, lungs, and spleen, and thus reduced the lethality of the plague infection. The dosage of GM spray-dried powders needed to achieve good prophylactic and therapeutic effects against the plague was less than that of the subcutaneous injection. In addition, potential advantages of the dry powder over the liquid nebulized formulations include: (i) powder formulations are easily actively inhaled by the patient, (ii) the powders are stable and minimally susceptible to environmental influences, and (iii) using excipients as bulking agents increased the total mass delivered. Therefore, we propose that GM spray-dried powders may provide an alternative and possibly a better treatment strategy against pneumonic plague.

Further study is warranted to facilitate clinical translation. Further optimization of the process parameters, such as inlet temperature and spray gas flow rate, is needed in subsequent studies to improve the powder yield and powder quality. Other powder preparation methods such as spray-freeze-drying can also be tried. In vivo pharmacokinetics studies are needed to determine local and systemic drug concentrations, etc. In addition, the synergy between the drug and the device is also very important for the human pulmonary delivery of dry powder. It is recommended to use widely accepted DPI devices, which are easy to operate without the assistance of trained medical personnel.

## 5. Conclusions

In this study, GM powders were prepared using spray-drying technology. The powders displayed good physical and chemical properties and met the requirements for human pulmonary inhalation. In a mouse model, inhalation therapy with GM spray-dried powders was an effective treatment for pneumonic plague. Taken together, GM administered by the inhalation route was significantly protective against *Y. pestis* when used as a post-exposure prophylaxis or treatment.

## Figures and Tables

**Table 1:** Factorial design parameters for optimization of GM spray-dried powders.

Independent Factors	Values Used, Actual (Coded Value)		
	Low (1)	Medium (2)	High (3)
A—Leu content (%, *w/w*)	5	10	15
B—inlet temperature (°C)	100	120	140
C—pump rate (%)	10	15	20
Dependent variables	Constraint		
Y	Minimize		

**Table 2:** Results of the orthogonal test for optimization.

Batch	A	B	C	d50 (μm)	Yield (%)	Y
A1B1C3	1	1	3	5.67	73.21	0.30
A1B2C2	1	2	2	5.18	74.20	0.23
A1B2C1	1	2	1	5.89	74.03	0.32
A2B3C1	2	3	1	4.76	76.11	0.16
A2B1C3	2	1	3	4.03	80.06	0.05
A2B1C2	2	1	2	4.10	78.47	0.07
A3B2C2	3	2	2	3.90	85.43	0.00
A3B3C1	3	3	1	4.41	81.25	0.09
A3B3C3	3	3	3	4.20	83.39	0.05
k1	0.85	0.42	0.57			
k2	0.28	0.55	0.30			
k3	0.14	0.30	0.40			
R	0.71	0.25	0.27			

**Table 3:** ANOVA results of the orthogonal test.

Factors	Sum of Squares	n	F	P
A	0.059	2	39.493	0.025
B	0.001	2	1.000	0.500
C	0.004	2	2.843	0.260
Error	0.001	2		

## Data Availability

Not applicable.
